# Association of platelet count with all‐cause mortality from acute respiratory distress syndrome: A cohort study

**DOI:** 10.1002/jcla.24378

**Published:** 2022-03-31

**Authors:** Rennv Wang, Haiwen Dai

**Affiliations:** ^1^ Emergency Department Affiliated Zhejiang Hospital of Zhejiang University School of Medical Hangzhou Zhejiang China

**Keywords:** acute respiratory distress syndrome, intensive care unit, mortality, platelet count

## Abstract

**Background:**

The purpose of this study was to investigate whether platelet count was associated with mortality in acute respiratory distress syndrome (ARDS) patients.

**Methods:**

We analyzed patients with ARDS from Multi‐parameter Intelligent Monitoring in Intensive Care Database III (MIMIC‐III). Platelet count was measured at the time of intensive care unit (ICU) admission. The cox proportional hazard model and subgroup analysis were used to determine the relationship between the platelet count and mortality of ARDS, as well as the consistency of its association. The primary outcome of this study was 365‐day mortality from the date of ICU admission.

**Result:**

This study enrolled a total of 395 critically ill patients with ARDS. After adjustment for age, gender and ethnicity, the multivariate cox regression model showed that the hazard ratios (HRs) (95% confidence intervals [CIs]) of platelet count <192 × 10^9^/L and >296 × 10^9^/L were 2.08 (1.43, 3.04) and 1.35 (0.91, 2.01), respectively, compared with the reference (192–296 ×10^9^/L). After adjusting for confounding factors, lower platelet count (<192 × 10^9^/L) was associated with increased mortality (adjusted HR, 1.71; 95% CI 1.06–2.76, *p* = 0.0284). However, there was no similar trend in the 30‐day (adjusted HR,1.02; 95% CI 0.54–1.94) or 90‐day (adjusted HR, 1.65; 95% CI 0.94–2.89) mortality. In the subgroup analysis, lower platelet count showed significant interactions with specific populations (*p* interaction = 0.0413), especially in patients with atrial fibrillation.

**Conclusion:**

Taken together, our analysis showed that platelet count is an independent predictor of mortality in critically ill patients with ARDS.

## Graphical abstract

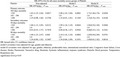

## 1 INTRODUCTION

Acute respiratory distress syndrome (ARDS) is characterized by noncardiogenic pulmonary edema and refractory hypoxemia, which is a serious complication associated with high mortality.^1^, ^2^, ^3^ Despite lung protection ventilation and intravenous steroids, many patients are still at risk of respiratory failure and death.^4^, ^5^ Previous studies have evaluated the risk factors for death in patients with ARDS.^6^, ^7^, ^8^ Given the severe clinical manifestations and poor prognosis of ARDS, there is need to identify more clinically meaningful predictors.

Platelet count is a set of platelet parameters that can be obtained by analysis of standard complete blood counts.^9^ Platelet count is mainly used for differential diagnosis of thrombotic diseases.^10^ Recent studies, however, have shown that platelets can be used as an independent prognostic indicator of mortality, and are associated with adverse outcomes in diseases such as sepsis,^11^ liver failure,^12^ multiple myeloma,^13^ and blood system diseases.^14^ In addition, low platelet count has been shown to be an independent risk factor for increased mortality in critically ill patients.^15^ Moreover, available data have demonstrated that platelets can regulate innate and adaptive immune responses,^16^ and can be used as a biomarker of both inflammatory and immune responses.^17^, ^18^, ^19^ Similarly, ARDS is also associated with immune and inflammatory responses.^20^, ^21^ Data on whether platelet count is associated with mortality of ARDS remains scant. There are no data on the relationship between platelet count and prognosis of patients with ARDS. Besides, it is unknown whether platelet count is a risk factor for ARDS in intensive care unit (ICU) patients. Here, we investigated the effect of platelet count in critically ill patients with ARDS.

## 2 MATERIALS AND METHOD

### 2.1 Sources of data

The present study used Multi‐parameter Intelligent Monitoring in Intensive Care Database III version 1.4 (MIMIC‐III v1.4^22^). This included more than 50,000 critically ill patients (medical, surgical, coronary care, and neonatal) admitted to Beth Israel Deaconess Hospital (Boston, MA, USA) from 2001 to 2012.^23^ The database was approved by the Institutional Review Board of the Beth Israel Deaconess Medical Center (Boston, MA) and the Massachusetts Institute of Technology (Cambridge, MA). In the current study, relevant clinical data were extracted, including patient demographics and laboratory test results.

### 2.2 Population selection criteria

The database contains a total of 58,976 critically ill patients and patients with ARDS age of 18 years or older were eligible for enrollment. The following symptoms were included in the Berlin criteria^24^, ^25^: acute onset, arterial partial pressure of oxygen (PaO_2_)/fraction of inspired oxygen (FiO_2_) <300 mmHg, positive end‐expiratory pressure ≥5 cm H_2_O, and on the first day of admission, the absence of heart failure and the presence of bilateral infiltrates on chest radiograph. The exclusion criteria of the patients from the current study included the following conditions: (1) no platelet count measurement during ICU hospitalization; (2) hematologic disease such as leukemia and myelodysplastic syndrome; (3) ICU hospital stay <48 h; and (4) missing >5% individual data.

### 2.3 Data extraction

The current study extracted the data using Structured Query Language (SQL) PostgreSQL (version 9.6). The content of the extracted data was demographic information, laboratory parameters, and clinical parameters. The extracted demographic information included: age, gender, ethnicity, systolic blood pressure, diastolic blood pressure (DBP), mean blood pressure, respiratory rate, heart rate, SPO_2_, and temperature.

The extracted comorbidities included congestive heart failure (CHF), atrial fibrillation, chronic liver disease, coronary artery disease (CAD), malignancy, and pneumonia. Furthermore, the extracted laboratory parameters included bicarbonate, creatinine, chloride, glucose, hematocrit, hemoglobin, potassium, platelet count (PLT), blood urea nitrogen (BUN), white blood cell (WBC), anion gap, albumin, sodium, bilirubin, lactate, activated partial thromboplastin time (APTT), prothrombin time (PT), and international normalized ratio (INR).

Sequential organ failure assessment (SOFA) and acute physiology score III (APSIII) were all calculated using physiological measurements and clinical information according to published recommendations and accepted formulae.^26^ The baseline data were extracted within 24 h after patient admission to the ICU. The primary outcome of the study was the 365‐day mortality from the date of ICU admission, whereas 30‐day and 90‐day mortality after ICU admission were the secondary outcomes.

### 2.4 PLT assessment

Venous blood samples were collected from subjects within 24 h of admission to the ICU. PLT is measured by medical instruments and expressed as 10^9^/L.

### 2.5 Statistical analysis

Baseline characteristics of all patients enrolled in the current study were stratified by platelet count tertiles. Furthermore, the chi‐square test was used for categorical variables and analysis of variance or Kruskal‐Wallis test for continuous variables to compare the groups. In addition, the continuous variables were expressed as mean ± standard deviation or medians, and categorical data were expressed as number or percentage. The cox proportional hazards model was used to determine the association between platelet count and hospital mortality as well as 365‐day all‐cause mortality for ARDS and the results were expressed as hazard ratios (HRs) with associated 95% confidence interval (CI).

Three models were run for each endpoint in the present study. All variables included in the multivariable model were selected according to their associations with the outcomes or a change in effect estimate of more than 10%. In model I, no covariates were adjusted. In model II, covariates were adjusted for age, gender, and ethnicity, whereas in model III, the covariates were further adjusted for DBP, temperature, respiratory rate, heart rate, CHF, chronic liver disease, stroke, pneumonia, vasoactive drug, systemic inflammatory response syndrome (SIRS), PT, and INR.

Furthermore, the stratification analyses were also performed to investigate whether the effect of platelets differed across various subgroups, including AFIB, CHF, CAD, CKD, liver disease, and vasoactive drug. The data were analyzed using the R software version 3.42. All statistical analyses were two‐sided and *p* < 0.05 was interpreted as statistically significant.

## 3 RESULT

### 3.1 Subject characteristics

A total of 395 patients who met the inclusion criteria were divided into three independent groups according to the level of platelet count. Individual characteristics and hematologic laboratory data of the study are presented in Table 1. The patients with a low platelet count value (Platelet count <192 × 10^9^/L) was more likely to be male and have lower heart rate, bicarbonate, glucose, and WBC than those with a high platelet count value (Platelet >296 × 10^9^/L).

**TABLE 1:** Characteristics of the study patients according to platelet count

Characteristics	Platelet count 10^9^/L			*p* value
	<192	192–296	>296	
*N*	130	132	133	
Age, years	63.2 ± 17.3	66.2 ± 17.7	64.7 ± 18.2	0.352
Gender, *n* (%)				0.004
Female	51 (39.2)	76 (57.6)	75 (56.4)	
Male	79 (60.8)	56 (42.4)	58 (43.6)	
Ethnicity, *n* (%)				0.953
White	93 (71.5)	93 (70.5)	98 (73.7)	
Black	12 (9.2)	15 (11.4)	13 (9.8)	
Other	25 (19.2)	24 (18.2)	22 (16.5)	
SBP, mmHg	115.1 ± 20.5	123.8 ± 19.7	116.0 ± 17.5	<0.001
DBP, mmHg	58.9 ± 15.5	61.3 ± 11.0	59.1 ± 11.0	0.013
Mean BP, mmHg	75.6 ± 15.6	81.0 ± 11.3	76.9 ± 11.6	<0.001
Respiratory rate, beats/min	22.4 ± 5.2	20.5 ± 4.5	21.9 ± 4.5	0.006
Heart rate, beats/min	90.2 ± 16.7	86.9 ± 17.2	92.2 ± 18.3	0.045
SPO_2_, %	96.4 ± 2.6	96.6 ± 2.2	96.7 ± 2.2	0.763
Temperature, ℃	37.0 ± 0.8	37.0 ± 0.8	36.9 ± 0.7	0.637
Comorbidities, *n* (%)				
CHF	18 (13.8)	23 (17.4)	19 (14.3)	0.678
AFIB	36 (27.7)	35 (26.5)	31 (23.3)	0.701
Chronic liver disease	23 (17.7)	3 (2.3)	4 (3.0)	<0.001
CAD	23 (17.7)	27 (20.5)	20 (15.0)	0.513
Malignancy	31 (23.8)	17 (12.9)	29 (21.8)	0.058
Pneumonia	68 (52.3)	62 (47.0)	77 (57.9)	0.205
Vasoactive drug, *n* (%)	55 (42.3)	42 (31.8)	46 (34.6)	0.188
Laboratory parameters				
Bicarbonate, mmol/l	20.8 ± 5.4	22.6 ± 4.7	21.4 ± 4.9	0.009
Creatinine, mEq/L	1.6 ± 1.5	1.3 ± 1.3	1.3 ± 1.5	0.013
Chloride, mmol/l	103.9 ± 7.6	101.7 ± 7.1	101.3 ± 6.9	<0.001
Glucose, mg/dl	136.7 ± 49.4	148.0 ± 47.6	156.1 ± 59.9	0.003
Hematocrit, %	29.9 ± 6.0	31.2 ± 6.1	30.0 ± 5.7	0.027
Hemoglobin, g/dl	10.1 ± 2.1	10.5 ± 2.2	10.1 ± 2.1	0.058
Potassium, mmol/l	3.8 ± 0.6	3.8 ± 0.6	3.9 ± 0.7	0.483
Platelet count, 10^9^/L	109.5 ± 52.1	204.9 ± 42.9	352.6 ± 127.2	<0.001
BUN, mg/dl	33.3 ± 24.1	27.1 ± 22.5	26.9 ± 25.0	0.004
WBC, 10^9^/L	11.7 ± 21.4	11.1 ± 5.4	15.4 ± 10.9	<0.001
Anion gap, mmol/L	13.6 ± 4.3	13.5 ± 3.3	14.3 ± 4.4	0.281
Albumin, g/L	2.9 ± 0.6	3.2 ± 0.7	2.9 ± 0.7	0.023
Sodium, mmol/L	137.7 ± 6.2	137.4 ± 5.9	136. ± 6.1	0.005
Bilirubin, mg/dl	3.9 ± 6.6	0.8 ± 1.1	0.8 ± 1.3	<0.001
Lactate, mmol/L	2.2 ± 2.2	1.7 ± 0.8	1.9 ± 1.1	0.493
APTT, s	35.3 ± 11.8	28.9 ± 8.6	29.4 ± 8.5	<0.001
PT, s	15.9 ± 4.3	14.2 ± 3.2	14.8 ± 3.3	<0.001
INR	1.5 ± 0.5	1.3 ± 0.6	1.4 ± 0.4	<0.001
30‐day mortality, *n* (%)	47 (36.2)	29 (22.0)	27 (20.3)	0.006
90‐day mortality, *n* (%)	59 (45.4)	36 (27.3)	44 (33.1)	0.007
365‐day mortality, *n* (%)	70 (53.8)	46 (34.8)	56 (42.1)	0.008

Note

Significance level *p* < 0.05.

Abbreviations: AFIB, atrial fibrillation; APTT, activated partial thromboplastin time; BUN, blood urea nitrogen; CAD, coronary artery disease; CHF, congestive heart failure; DBP, diastolic blood pressure; INR, international normalized ratio; PT, prothrombin time; SBP, systolic blood pressure; SPO_2_, blood oxygen saturation; WBC, white blood cell.

Patients with a low platelet count also had higher creatinine, respiratory rate, chloride, sodium, bilirubin, BUN, APTT, and PT than those with a high platelet count. The INR was significantly higher in the group of high platelet count. Moreover, low platelet count was more frequent in patients with liver disease as compared with the rest of the participants.

### 3.2 Platelet count levels and long‐term mortality

It was found that low platelet count was associated with an increased risk of hospital mortality and 365‐day all‐cause mortality in patients with ARDS (Table 2). Results of the multivariate Cox regression analysis for 365‐day mortality, HRs (95% CIs) of platelet count <192 × 10^9^/L and >296 × 10^9^/L as compared with the reference level (192–296 × 10^9^/L), were 1.82 (1.25, 2.64) and 1.25 (0.84, 1.84), respectively.

**TABLE 2:** HR (95% CIs) for all‐cause mortality across groups of platelet count

Platelet count 10^9^/L	Non‐adjusted		Model I		Model II	
	HR (95% CIs)	*p* value	HR (95% CIs)	*p* value	HR (95% CIs)	*p* value
Primary outcome						
365‐day mortality						
<192	1.82 (1.25, 2.64)	0.0017	2.08 (1.43, 3.04)	0.0001	1.71 (1.06, 2.76)	0.0284
192–296	1.0		1.0		1.0	
>296	1.25 (0.84, 1.84)	0.2704	1.35 (0.91, 2.01)	0.1305	1.21 (0.76, 1.93)	0.4160
Secondary outcomes						
30‐day mortality						
<192	1.80 (1.13, 2.85)	0.0131	1.99 (1.24, 3.19)	0.0043	1.02 (0.54, 1.94)	0.9484
192–296	1.0		1.0		1.0	
>296	0.93 (0.55, 1.57)	0.7878	0.98 (0.58, 1.67)	0.9502	0.68 (0.35, 1.32)	0.2541
90‐day mortality						
<192	1.90 (1.25, 2.87)	0.002	2.19 (1.43, 3.34)	0.0003	1.65 (0.94, 2.89)	0.0791
192–296	1.0		1.0		1.0	
>296	1.24 (0.80, 1.92)	0.3407	1.34 (0.86, 2.09)	0.1951	1.05 (0.61, 1.82)	0.8616

Note

Model I covariates were adjusted for age, gender, and ethnicity.

Model II covariates were adjusted for age; gender; ethnicity; prothrombin time; international normalized ratio; congestive heart failure; liver disease; stroke; pneumonia; vasoactive drug; heartrate; systemic inflammatory response syndrome; diastolic blood pressure; temperature; respiratory rate.

Significance level *p* < 0.05.

Abbreviations: CI, confidence interval; HR, hazard ratio.

When adjusted for age, ethnicity, and gender, results of the present study were as follows: HRs (95% CIs) of Platelet count <192 × 10^9^/L and >296 × 10^9^/L as compared with the reference level (192–296 × 10^9^/L), were 2.08 (1.43, 3.04) and 1.35 (0.91, 2.01), respectively. Moreover, after adjustments for age, gender, ethnicity, PT, INR, CHF, chronic liver disease, stroke, pneumonia, vasoactive drug, heart rate, SIRS, DBP, temperature, and respiratory rate, the lower platelet count (<192 × 10^9^/L) was also associated with an increased risk of mortality (adjusted HR, 1.71; 95% CI 1.06–2.76, *p* = 0.0284).

### 3.3 Platelet count levels and 30‐day and 90‐day mortality

For the secondary outcomes of 30‐day and 90‐day mortality, the results of multivariate analysis showed that the HR (95% CIs) of low platelet count (<192 × 10^9^/L) was 1.80 (1.13, 2.85) and 1.90 (1.25, 2.87), respectively. After adjustment for age, gender, and ethnicity, the HR (95% CIs) of the low group was 1.99 (1.24, 3.19) and 2.19 (1.43, 3.34), respectively. After further adjustment of some complicated factors, for 30‐day (adjusted HR, 1.02; 95% CI 0.54–1.94) and 90‐day all‐cause mortality (adjusted HR, 1.65; 95% CI 0.94–2.89), the association between low platelet count and all‐cause mortality in patients with ARDS in ICU did not have statistical significance.

### 3.4 Subgroup analyses

Subgroup analyses were also performed in the present study to examine the consistency of association between platelet count and risk of 365‐day hospital mortality in patients with ARDS. It included CHF, CAD, AFIB, liver disease, CKD, and vasoactive drug in the analyses (Table 3). In model I, patients with AFIB had a significantly higher risk of hospital mortality with lower platelet count (HR 1.27, 95% CI 0.68–2.37). Moreover, the platelet count showed significant interactions with patients with AFIB (*p* interaction = 0.0413) for 365‐day mortality of ARDS in ICU.

**TABLE 3:** Cox proportional hazards analysis of the ability of platelet count to predict all‐cause mortality

	Platelet count, 10^9^/L			*p* interaction
	<192	192–296	>296	
CHF				0.3467
No	2.18 (1.44, 3.30)	1.0	1.24 (0.80, 1.91)	
Yes	1.99 (0.65, 6.12)	1.0	1.73 (0.60, 5.00)	
AFIB				0.0413
No	2.90 (1.77, 4.74)	1.0	1.50 (0.90, 2.49)	
Yes	1.27 (0.68, 2.37)	1.0	1.33 (0.70, 2.51)	
Liver disease				0.9308
No	1.84 (1.23, 2.75)	1.0	1.38 (0.92, 2.05)	
Yes	2.66 (0.31, 22.71)	1.0	1.12 (0.07, 17.22)	
CAD				0.6445
No	2.44 (1.57, 3.78)	1.0	1.45 (0.92, 2.27)	
Yes	1.37 (0.62, 3.03)	1.0	1.09 (0.47, 2.52)	
Vasoactive drug				0.2778
No	2.55 (1.48, 4.38)	1.0	1.79 (1.06, 3.02)	
Yes	1.54 (0.88, 2.68)	1.0	0.89 (0.48, 1.68)	
CKD				0.5900
No	1.93 (1.28, 2.90)	1.0	1.23 (0.81, 1.88)	
Yes	1.67 (0.46, 6.03)	1.0	4.61 (1.11, 19.11)	0.3467

Note

Model I covariates were adjusted for age, gender, and ethnicity.

Significance level *p* < 0.05.

Abbreviations: AFIB: atrial fibrillation; CAD: coronary artery disease; CHF: congestive heart failure; CKD: chronic kidney disease.

## 4 DISCUSSION

It was evident that low platelet count is an independent risk factor for 365‐day all‐cause mortality in patients with ARDS in the ICU. In addition, platelet count showed a significant interaction with AFIB in the 365‐day mortality of patients with ARDS in ICU. Furthermore, recent studies have shown that platelet count is a new predictor of multiple adverse outcomes.^27^, ^28^, ^29^, ^30^, ^31^ The current study also indicated that lower platelet count was an independent predictor of mortality in patients with ARDS in ICU.

The ARDS is a life‐threatening complication with high mortality and is a systemic inflammatory response syndrome.^1^, ^20^, ^32^ It is associated with multiple organ disorders and systemic inflammation.^2^, ^20^ In several previous studies, some biomarkers have been used to predict the prognosis of ARDS.^33^, ^34^, ^35^ However, prognostic indicators of ARDS are limited because a single mechanism cannot predict the outcome of a complex syndrome like ARDS.^35^, ^36^, ^37^

Platelet count is a low cost and easy to obtain routine item of laboratory examinations. A lot of research studies have shown that platelet count is a reliable predictor of various diseases and organ dysfunction.^17^, ^38^ Some studies have found a link between platelet count and platelet count changes in inflammatory markers.^38^, ^39^ Furthermore, platelets can limit the growth of bacteria, affect the recruitment and function of white blood cells, as well as cause a series of changes in cytokines.^40^, ^41^ Therefore, systemic inflammatory response and multiple organ failure may help explain the potential link between platelet count and mortality in patients with ARDS in the ICU, but the mechanisms are still not clear. The low platelet count could be caused by thrombocytopenia or DIC and both are common complications among critically ill patients and are independent risk factors affecting prognosis.^38^, ^42^, ^43^, ^44^

It has been noted that at the setting of severe disease such as sepsis, low platelet count leads to platelet count aggregation, platelet‐leukocyte complex formation, and release of molecules that enhance inflammation as well as cell adhesion.^20^, ^44^, ^45^ Increases in pro‐inflammatory factors and adhesion molecules have a negative impact on the survival of patients.^45^, ^46^ Platelet‐mediated thrombosis and increased permeability of the endothelial‐capillary barrier both reveal a close link between platelet count and ARDS mortality.^10^, ^47^, ^48^

To the best of our knowledge, the current research was the first study to fully determine the relationship between platelet count and mortality in patients with ARDS in the ICU. In addition, mortality was chosen as the main outcome.

However, the present study also had some limitations. First, a single‐center retrospective design was used with the influence of selection bias. Second, platelet count was measured in patients only upon admission to the ICU and changes in platelet count during ICU hospitalization were not assessed. Third, the number of patients selected was not large. Finally, this study did not consider interaction or nonlinearity for the relationship between covariates and outcome when constructing the model. Ensemble modeling may be needed to address these questions fully.^49^

## 5 CONCLUSION

In conclusion, it was found that platelet count is a novel and independent predictor of mortality in patients with ARDS and in the ICU. In addition, lower platelet count showed significant interactions with specific populations and especially in patients with atrial fibrillation. However, there is need for more extensive prospective studies and long‐term follow‐up to determine the relationship between platelet count and poor prognosis in patients with cancer.

## DISCLOSURE

The funders of the project were not involved in study design; in the collection; in the data analysis; or in the writing of the report and publication.

## CONFLICT OF INTEREST

The authors declare that they have no competing interests.

## Data Availability

The data that support the findings of this study are available on request from the Haiwen Dai (E‐mail: m13958001551@21cn.com).

## References

[1] Villar J , Blanco J , Kacmarek RM . Current incidence and outcome of the acute respiratory distress syndrome. Curr Opin Crit Care. 2016;22(1):1‐6. 26645551 10.1097/MCC.0000000000000266

[2] Neto AS , Barbas CSV , Simonis FD , et al. Epidemiological characteristics, practice of ventilation, and clinical outcome in patients at risk of acute respiratory distress syndrome in intensive care units from 16 countries (PRoVENT): an international, multicentre, prospective study. Lancet Resp Med. 2016;4(11):882‐893. 10.1016/S2213-2600(16)30305-8 27717861

[3] Villar J , González‐Martín J , Ambrós A , et al. Stratification for Identification of Prognostic Categories In the Acute RESpiratory Distress Syndrome (SPIRES) Score. Crit Care Med. 2021;49(10):e920–e930. doi:10.1097/ccm.0000000000005142 34259448

[4] Rozencwajg S , Pilcher D , Combes A , Schmidt M . Outcomes and survival prediction models for severe adult acute respiratory distress syndrome treated with extracorporeal membrane oxygenation. Crit Care. 2016;20:10. 27919283 10.1186/s13054-016-1568-y PMC5139100

[5] Brower RG , Matthay MA , Morris A , et al. Ventilation with lower tidal volumes as compared with traditional tidal volumes for acute lung injury and the acute respiratory distress syndrome. N Engl J Med. 2000;342(18):1301‐1308. 10793162 10.1056/NEJM200005043421801

[6] Navarrete‐Navarro P , Rodriguez A , Reynolds N , et al. Acute respiratory distress syndrome among trauma patients: trends in ICU mortality, risk factors, complications and resource utilization. Intensive Care Med. 2001;27(7):1133‐1140. 11534560 10.1007/s001340100955

[7] Hughes CG , Weavind L , Banerjee A , Mercaldo ND , Schildcrout JS , Pandharipande PP . Intraoperative risk factors for acute respiratory distress syndrome in critically ill patients. Anesth Analg. 2010;111(2):464‐467. 20418537 10.1213/ANE.0b013e3181d8a16a

[8] de Prost N , Pham T , Carteaux G , et al. Etiologies, diagnostic work‐up and outcomes of acute respiratory distress syndrome with no common risk factor: a prospective multicenter study. Ann Intensive Care. 2017;7:12. 28631088 10.1186/s13613-017-0281-6 PMC5476531

[9] Bessman JD . Evaluation of automated whole‐blood platelet counts and particle sizing. Am J Clin Pathol. 1980;74(2):157‐162. 7405893 10.1093/ajcp/74.2.157

[10] Schafer AI , Handin RI . The role of platelets in thrombotic and vascular disease. Prog Cardiovasc Dis. 1979;22(1):31‐52. 379914 10.1016/0033-0620(79)90002-1

[11] Xu Y , Jin X , Shao X , Zheng F , Zhou H . Valuable prognostic indicators for severe burn sepsis with inhalation lesion: age, platelet count, and procalcitonin. Burns Trauma. 2018;6:29. 30397617 10.1186/s41038-018-0132-1 PMC6205790

[12] Jie Y , Gong J , Xiao C , et al. Low platelet to white blood cell ratio indicates poor prognosis for acute‐on‐chronic liver failure. Biomed Res Int. 2018;5. doi:10.1155/2018/7394904 PMC5964479 29854786

[13] Wongrakpanich S , George G , Chaiwatcharayut W , et al. The prognostic significance of neutrophil‐to‐lymphocyte and platelet‐to‐lymphocyte ratios in patients with multiple myeloma. J Clin Lab Anal. 2016;30(6):1208‐1213. 27239981 10.1002/jcla.22004 PMC6807214

[14] Zlobina KE , Guria GT . Platelet activation risk index as a prognostic thrombosis indicator. Sci Rep. 2016;6:6. 27461235 10.1038/srep30508 PMC4962318

[15] Moreau D , Timsit J‐F , Vesin A , et al. Platelet count decline ‐ an early prognostic marker in critically ill patients with prolonged ICU stays. Chest. 2007;131(6):1735‐1741. 17475637 10.1378/chest.06-2233

[16] Sprague DL , Elzey BD , Crist SA , Waldschmidt TJ , Jensen RJ , Ratliff TL . Platelet‐mediated modulation of adaptive immunity: unique delivery of CD154 signal by platelet‐derived membrane vesicles. Blood. 2008;111(10):5028‐5036. 18198347 10.1182/blood-2007-06-097410 PMC2384131

[17] Gawaz M , Dickfeld T , Bogner C , Fateh‐Moghadam S , Neumann FJ . Platelet function in septic multiple organ dysfunction syndrome. Intensive Care Med. 1997;23(4):379‐385. 9142575 10.1007/s001340050344

[18] Matowicka‐Karna J , Kamocki Z , Polinska B , Osada J , Kemona H . Platelets and inflammatory markers in patients with gastric cancer. Clin Dev Immunol. 2013;2013:1–6. 10.1155/2013/401623 PMC3608177 23554823

[19] Topal E , Celiksoy MH , Catal F , Karakoc HTE , Karadag A , Sancak R . The platelet parameters as inflammatory markers in preschool children with atopic eczema. Clin Lab. 2015;61(5–6):493‐496. 26118181

[20] Bhatia M , Moochhala S . Role of inflammatory mediators in the pathophysiology of acute respiratory distress syndrome. J Pathol. 2004;202(2):145‐156. 14743496 10.1002/path.1491

[21] Capelozzi VL , Allen TC , Beasley MB , et al. Molecular and immune biomarkers in acute respiratory distress syndrome a perspective from members of the Pulmonary Pathology Society. Arch Pathol Lab Med. 2017;141(12):1719‐1727. 28613912 10.5858/arpa.2017-0115-SA

[22] Sun H , Que J , Peng Y , et al. The neutrophil‐lymphocyte ratio: a promising predictor of mortality in coronary care unit patients — a cohort study. Int Immunopharmacol. 2019;74:105692. 31228818 10.1016/j.intimp.2019.105692

[23] Johnson AEW , Pollard TJ , Shen LU , et al. MIMIC‐III, a freely accessible critical care database. Sci Data. 2016;3:9. 10.1038/sdata.2016.35 PMC4878278 27219127

[24] Saha R , Assouline B , Mason G , Douiri A , Summers C , Shankar‐Hari M . Impact of differences in acute respiratory distress syndrome randomised controlled trial inclusion and exclusion criteria: systematic review and meta‐analysis. Br J Anaesth. 2021;127(1):85‐101. 33812666 10.1016/j.bja.2021.02.027 PMC9768208

[25] Riviello ED , Kiviri W , Twagirumugabe T , et al. Hospital incidence and outcomes of the acute respiratory distress syndrome using the Kigali modification of the Berlin definition. Am J Respir Crit Care Med. 2016;193(1):52‐59. 26352116 10.1164/rccm.201503-0584OC

[26] Herwanto V , Shetty A , Nalos M , et al. Accuracy of quick sequential organ failure assessment score to predict sepsis mortality in 121 studies including 1,716,017 individuals. A systematic review and meta‐analysis. Crit Care Explor. 2019;1(9):e0043. 32166285 10.1097/CCE.0000000000000043 PMC7063937

[27] Liu W‐Y , Lin S‐G , Wang L‐R , et al. Platelet‐to‐lymphocyte ratio a novel prognostic factor for prediction of 90‐day outcomes in critically ill patients with diabetic ketoacidosis. Medicine (Baltimore). 2016;95(4):7. 10.1097/MD.0000000000002596 PMC5291578 26825908

[28] Goncalves SC , Labinaz M , Le May M , et al. Usefulness of mean platelet volume as a biomarker for long‐term outcomes after percutaneous coronary intervention. Am J Cardiol. 2011;107(2):204‐209. 21129717 10.1016/j.amjcard.2010.08.068

[29] Sezgi C , Taylan M , Kaya H , et al. Alterations in platelet count and mean platelet volume as predictors of patient outcome in the respiratory intensive care unit. Clin Respir J. 2015;9(4):403‐408. 24725778 10.1111/crj.12151

[30] Quan W , Chen Z , Yang X , et al. Mean platelet volume/platelet count ratio as a predictor of 90‐day outcome in large artery atherosclerosis stroke patients. Int J Neurosci. 2017;127(11):1019‐1027. 28270030 10.1080/00207454.2017.1296438

[31] Lin C‐Y , Chang C‐Y , Sun C‐H , et al. Platelet count and early outcome in patients with spontaneous cerebellar hemorrhage: a retrospective study. PLoS One. 2015;10(3):9. 10.1371/journal.pone.0119109 PMC4364557 25781880

[32] Villar J , Sulemanji D , Kacmarek RM . The acute respiratory distress syndrome: incidence and mortality, has it changed? Curr Opin Crit Care. 2014;20(1):3‐9. 24309954 10.1097/MCC.0000000000000057

[33] Terpstra ML , Aman J , Amerongen GPV , Groeneveld ABJ . Plasma biomarkers for acute respiratory distress syndrome: a systematic review and meta‐analysis. Crit Care Med. 2014;42(3):691‐700. 24158164 10.1097/01.ccm.0000435669.60811.24

[34] Binnie A , Tsang JLY , dos Santos CC . Biomarkers in acute respiratory distress syndrome. Curr Opin Crit Care. 2014;20(1):47‐55. 24296379 10.1097/MCC.0000000000000048

[35] Garcia‐Laorden MI , Lorente JA , Flores C , Slutsky AS , Villar J . Biomarkers for the acute respiratory distress syndrome: how to make the diagnosis more precise. Ann Transl Med. 2017;5(14):283. 28828358 10.21037/atm.2017.06.49 PMC5537109

[36] Mekontso Dessap A , Boissier F , Charron C , et al. Acute cor pulmonale during protective ventilation for acute respiratory distress syndrome: prevalence, predictors, and clinical impact. Intensive Care Med. 2016;42(5):862‐870. 26650055 10.1007/s00134-015-4141-2

[37] Wang CY , Calfee CS , Paul DW , et al. One‐year mortality and predictors of death among hospital survivors of acute respiratory distress syndrome. Intensive Care Med. 2014;40(3):388‐396. 24435201 10.1007/s00134-013-3186-3 PMC3943651

[38] Zhang J , Zhang HY , Li J , Shao XY , Zhang CX . The elevated NLR, PLR and PLT may predict the prognosis of patients with colorectal cancer: a systematic review and meta‐analysis. Oncotarget. 2017;8(40):68837‐68846. 28978160 10.18632/oncotarget.18575 PMC5620300

[39] Wei Y , Wang Z , Su LI , et al. Platelet count mediates the contribution of a genetic variant in LRRC16A to ARDS risk. Chest. 2015;147(3):607‐617. 25254322 10.1378/chest.14-1246 PMC4347530

[40] Hara T , Shimizu K , Ogawa F , et al. Platelets control leukocyte recruitment in a murine model of cutaneous arthus reaction. Am J Pathol. 2010;176(1):259‐269. 20008131 10.2353/ajpath.2010.081117 PMC2797888

[41] Xiang B , Zhang G , Guo L , et al. Platelets protect from septic shock by inhibiting macrophage‐dependent inflammation via the cyclooxygenase 1 signalling pathway. Nature Communications. 2013;4(1). doi:10.1038/ncomms3657 PMC4217311 24150174

[42] Daugirdas JT , Bernardo AA . Hemodialysis effect on platelet count and function and hemodialysis‐associated thrombocytopenia. Kidney Int. 2012;82(2):147‐157. 22592187 10.1038/ki.2012.130

[43] Qu M , Liu Q , Zhao H‐G , et al. Low platelet count as risk factor for infections in patients with primary immune thrombocytopenia: a retrospective evaluation. Ann Hematol. 2018;97(9):1701‐1706. 29777278 10.1007/s00277-018-3367-9 PMC6097778

[44] Reilly JP , Christie JD . Linking genetics to ARDS pathogenesis the role of the platelet. Chest. 2015;147(3):585‐586. 25732436 10.1378/chest.14-2701 PMC4364315

[45] Ware LB , Koyama T , Zhao Z , et al. Biomarkers of lung epithelial injury and inflammation distinguish severe sepsis patients with acute respiratory distress syndrome. Crit Care. 2013;17(5):7. 10.1186/cc13080 PMC4056313 24156650

[46] Agouridakis P , Kyriakou D , Alexandrakis MG , et al. The predictive role of serum and bronchoalveolar lavage cytokines and adhesion molecules for acute respiratory distress syndrome development and outcome. Respir Res. 2002;3(1):9. 12537603 10.1186/rr193 PMC150513

[47] Fernández‐Martín L , Marcos‐Ramiro B , Bigarella CL , et al. Crosstalk between reticular adherens junctions and platelet endothelial cell adhesion molecule‐1 regulates endothelial barrier function. Arterioscler Thromb Vasc Biol. 2012;32(8):E90‐U141. 22723439 10.1161/ATVBAHA.112.252080

[48] Mei H , Campbell JM , Paddock CM , et al. Regulation of endothelial cell barrier function by antibody‐driven affinity modulation of platelet endothelial cell adhesion molecule‐1 (PECAM‐1). J Biol Chem. 2014;289(30):20836‐20844. 24936065 10.1074/jbc.M114.557454 PMC4110291

[49] Zhang Z , Chen L , Xu P , Hong Y . Predictive analytics with ensemble modeling in laparoscopic surgery: a technical note. 2022. Laprosc Endosc Robot Surg. 2022;5:25–34.

